# Another Case of De Novo 3q26.33q27.3 Microdeletion and Its Medicolegal Sequel

**DOI:** 10.1155/2018/1909056

**Published:** 2018-02-07

**Authors:** Ugo Indraccolo, Salvatore Renato Indraccolo, Piergiorgio Fedeli

**Affiliations:** ^1^Complex Operative Unit of Obstetrics and Gynecology, “Alto Tevere” Hospital of Città di Castello, ASL 1 Umbria, Via Luigi Angelini 10, 06012 Città di Castello, Italy; ^2^Department of Gynecological, Obstetrical, and Urological Sciences, Policlinico Umberto I, “Sapienza” University of Rome, Via del Policlinico 155, 00161 Roma, Italy; ^3^School of Law, Legal Medicine Section, University of Camerino, Via A. D'Accorso 16, 62032 Camerino, Italy

## Abstract

A new case of a de novo 3q26.33q27.3 microdeletion is reported. The fetus had a sonographically undiagnosable polymalformative syndrome. The case highlights the difficulties of echographic diagnosis of such syndromes and leads to reflection on the difficulties of appropriate counselling in cases of uncertainty.

## 1. Introduction

Newborn cases with the 3q26.33q27.3 microdeletion are very uncommon. To the best of our knowledge, Bouman et al. reported the last case of the 3q26.33q27.3 microdeletion [1]. Those authors reviewed similar cases of microdeletion on the q arm of chromosome 3, focusing on clinical features of newborns with these chromosomal abnormalities. They found seven additional cases, with the first case reported in 1984 [2].

Therefore, it seems of interest to report another case of 3q26.33q27.3 microdeletion, which was referred to the authors during a medicolegal assessment because of failure of intrauterine diagnosis. The authors provide the following information on the case based on their medicolegal documents. The parents gave permission to report the case from a medicolegal point of view.

## 2. Case

The story of the case begins in 2008 with the first echographic assessment performed on the mother who was 23 years old, II gravida 0 para, at 10 weeks and 2 days of amenorrhea. Based on crown-rump-length, the gestational age was given as 7 weeks and 4 days. Patients underwent routine lab examinations and another echographic assessment at 10 weeks and 4 days, in which no abnormalities were recorded. Again, routine lab examinations were suggested.

At 20 weeks and 1 day, echographic examination was performed. Biometric measurements were performed, and no abnormalities were detected on some fetal morphologic features. Biparietal distance (BPD), cranial circumference (CC), and abdominal circumference (AC) were found to be below the 5th centile. The femur length (FL) was at the 50th centile. Routine lab examinations were suggested. Fetal screening of any kind was not suggested. However, based on the low centiles recorded, a second opinion about the pregnancy was requested by the patient herself. She underwent another echographic assessment at 20 weeks and 5 days performed by another gynecologist. Low centiles were confirmed, and another more accurate echographic examination was scheduled in the following week. Subsequently, at 21 weeks and 5 days, low centiles of BPD, CC, and AC were confirmed, while FL and humerus length (HL) were found to be at the 50% centile. Complete assessment of the morphology was taken, without additional abnormalities. Remarkably, orbital cavities with crystallines were visualized. General counselling about the low centiles of the fetal head was provided, along with limitations of the echographic examination of the fetal morphology. The isolate low centiles of the head were put in relationship with the anthropometric characteristics of the parents, although the parents did not have small heads (the head circumference of the parents was not recorded). Nevertheless, another echographic check of the fetal morphology and growth after six or seven weeks was suggested. Approximately one month later (24 weeks and 6 days), another echographic scan was performed by the first gynecologist along with a check of some anatomical features of the fetus. Information on the orbital cavities was not reported. The previous findings were confirmed, with regular growth on the 5th centile of the head and the abdomen (asymmetric growth) and a 60th centile femur length. Routine lab examinations were suggested. Two additional 3rd trimester echographic checks, the first one performed by the first gynecologist (28 weeks and 4 days) and the second one performed by another (36 weeks), confirmed the 5th centile growth of the head and abdomen, asymmetric growth, and regular morphology of some anatomic features of the fetus. The 28-week sonographic check found a 20th centile femur length, while the 36-week check found an uncertain 80th centile value of femur length. No description of the orbital cavities was reported, and a podalic presentation was diagnosed. At 39 weeks and 4 days, a planned cesarean section was performed because of persistent podalic presentation. A small for gestational age female fetus was born, classified as Apgar 7–9 and weighing 1895 g. The newborn had microphthalmia, with small eyeballs, palpebral fissures, diastase of the first finger of both hands and feet (sandal sign), ambiguous genitalia, convoluted helix, deafness, small thickness of the corpus callosum (no agenesis was recorded), microgyria, microcephalia, and mild tricuspid dysplasia. A standard chromosomal assessment did not find any abnormalities.

Two months later, the baby was treated for a high respiratory tract infection. Difficulties in oral feeding and low growth rate were also found. Moreover, she often suffered from constipation. A CGH-Array (3/3/10) with a resolution of 250 kilobases found the de novo 3q26.33q27.3 microdeletion, with a loss of approximately 7.6 megabases. Severe mental delay was determined in the child.

Based on the severe consequences of this polymalformative syndrome, the parents wanted to know if everything was correctly done during the pregnancy and initiated a litigation. The case was difficult to treat from a medicolegal point of view because an extremely rare malformative syndrome was faced. The key points of the litigation were as follows: (i) Was the polymalformative syndrome able to be diagnosed in uterus? (ii) Were the echographic examinations correctly performed?

Regarding the latter, during the litigation, it was ascertained that the Italian practice guidelines for echographic examinations in pregnancy [3–5] were overall followed as should be done in case of a normal pregnancy. However, was it a normal pregnancy? At the second trimester, in at least one examination, all the morphologic assessments of the fetus were correctly carried out and resulted in normality, with the only exception being the findings of isolated severe microcephalia and small size for the gestational age. It is unclear if the crystallines were visible at the time, as Italian practice guidelines suggest to check. It seems unlikely that the crystallines were developed in this case of severe microphthalmia. It is also unclear if false imaging of orbital cavities could be seen by gynecologists in such a rare condition, leading them to believe that crystallines developed. Bouman et al. [1], in their case, clearly stated that the microphthalmia was not diagnosed echographically and that the pregnancy was interrupted because multiple other malformations were diagnosed sonographically. Additionally, in Bouman et al.'s case [1], the amniocentesis was negative. In the present case, both the isolated microcephalia and the small size for gestational age trend of growth (with asymmetric pattern) were labeled as normal. The fetal growth was carefully checked, as recommended for a normal pregnancy. This behavior would be appropriate if a healthy baby had been born. However, the echographic diagnosis of the syndrome appears to be very challenging; therefore, the only flaw in the care of the pregnancy was the lack of counselling on the outcome of abnormal microcephalia. The parents did not understand that although isolated microcephalia is benign in nearly the majority of cases, it can sometimes be linked to mental delay [6]. This delay can occur even if microcephalia is the only finding during morphological assessment of the fetus. Even in the case of asymmetric growth on low centiles, the gynecologists did not take into account the fact that the microcephalia could be abnormal and thus did not provide counselling on this.

During the judgment, however, the liability of the gynecologists due to the lack of counselling was not clearly proven, and the verdict was in favor of the gynecologists.

## 3. Discussion

The case presented here is of interest from both a clinical point of view and a medicolegal point of view. For clinicians, it is of interest to confirm that the de novo 3q26.33q27.3 microdeletion seems to typically have ocular absence or severe microphthalmia and microcephalia, along with mental delay [1]. Moreover, it seems difficult to diagnose the 3q26.33q27.3 microdeletion syndrome in uterus in the absence of morphological abnormalities detectable by echography. Among these, it seems very unlikely to diagnose the ocular abnormalities at the second-trimester echographic examination.

The overall sensitivity of routine second-trimester echographic screening for fetal malformations has been reported to be low [3, 7–13], generating some concerns about its overall effectiveness [14]. Currently, it is acknowledged that echographic assessment of fetal malformations should be performed by trained personnel with good equipment and more than once during pregnancy [3, 14]. All these conditions do not seem to match the goal of a “routine screening.” The parents, however, await clear answers from what they acknowledge as a “routine screening.”

The medicolegal aspects of this case led to reflection on the key role of counselling in the case of an uncommon unfavorable outcome of a finding that can be more likely benign during the “routine” echographic screening of fetal malformations. It seems that gynecologists fell into error because they truly believed that the microcephalia was normal and did not want to frighten the parents in the case of a normal finding. They considered the pregnancy to be a normal, healthy pregnancy.

In the case of a very rare and undiagnosable disease, anyone could lack counselling. The impact of the appropriate counselling should be considered when unnecessary fear and anxiety are provoked in the case of a more common normal finding. The disappointment of parents should be avoided in the case of devastating consequences of a nondiagnosis and unnecessary anxiety because a normal finding cannot be proven. Theoretically, this anxiety could be the cause of an unnecessary pregnancy termination. How can physician counsel about his uncertainty?
